# 4-[2-(4-But­oxy­phen­yl)ethen­yl]-1-methyl­pyridinium tosylate

**DOI:** 10.1107/S1600536813009616

**Published:** 2013-04-13

**Authors:** M. Krishna Kumar, S. Mabel Margret, G. Chakkaravarthi, D. Velmurugan, R. Mohan Kumar

**Affiliations:** a Department of Physics, Presidency College, Chennai 600 005, India; b Department of Physics, CPCL Polytechnic College, Chennai 600 068, India; c Centre of Advanced Study in Crystallography and Biophysics, University of Madras, Maraimalai Campus, Chennai 600 025, India

## Abstract

In the title mol­ecular salt, C_18_H_22_NO^+^·C_7_H_7_O_3_S^−^, the dihedral angle between the aromatic rings in the cation is 10.00 (9)°; its alkyl side chain adopts an extended conformation. In the crystal, weak C—H⋯O and π–π [centroid–centroid distance = 3.7658 (17) Å] inter­actions link the components, generating a three-dimensional network.

## Related literature

For mol­ecular compounds with non-linear optical properties, see: Nalwa & Miyata (1997 ▶). For related structures, see: Krishnakumar *et al.* (2012 ▶); Sivakumar *et al.*(2012 ▶).
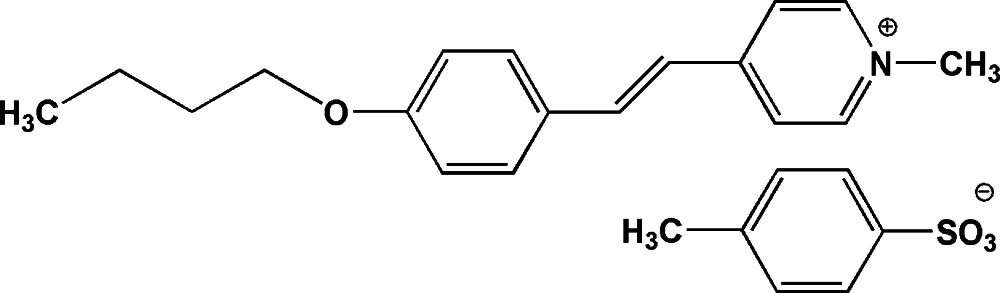


## Experimental

### 

#### Crystal data

C_18_H_22_NO^+^·C_7_H_7_O_3_S^−^
*M* *_r_* = 439.55
Monoclinic, 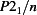

*a* = 9.0884 (6) Å
*b* = 6.4559 (5) Å
*c* = 39.827 (3) Å
β = 95.404 (3)°
*V* = 2326.4 (2) Å^3^
*Z* = 4
Mo *K*α radiation
μ = 0.17 mm^−1^
*T* = 295 K
0.28 × 0.24 × 0.20 mm

#### Data collection

Bruker Kappa APEXII CCD diffractometer
Absorption correction: multi-scan (*SADABS*; Sheldrick, 1996 ▶) *T*_min_ = 0.954, *T*_max_ = 0.967
20608 measured reflections
5629 independent reflections
4258 reflections with *I* > 2σ(*I*)
*R*_int_ = 0.028

#### Refinement

*R*[*F* ^2^ > 2σ(*F* ^2^)] = 0.069
*wR*(*F* ^2^) = 0.160
*S* = 1.16
5629 reflections
291 parameters
2 restraints
H atoms treated by a mixture of independent and constrained refinement
Δρ_max_ = 0.23 e Å^−3^
Δρ_min_ = −0.39 e Å^−3^

### 

Data collection: *APEX2* (Bruker, 2004 ▶); cell refinement: *SAINT* (Bruker, 2004 ▶); data reduction: *SAINT*; program(s) used to solve structure: *SHELXS97* (Sheldrick, 2008 ▶); program(s) used to refine structure: *SHELXL97* (Sheldrick, 2008 ▶); molecular graphics: *PLATON* (Spek, 2009 ▶); software used to prepare material for publication: *SHELXL97*.

## Supplementary Material

Crystal structure: contains datablock(s) global, I. DOI: 10.1107/S1600536813009616/hb7067sup1.cif

Structure factors: contains datablock(s) I. DOI: 10.1107/S1600536813009616/hb7067Isup2.hkl

Supplementary material file. DOI: 10.1107/S1600536813009616/hb7067Isup3.cml

Additional supplementary materials:  crystallographic information; 3D view; checkCIF report

## Figures and Tables

**Table 1:** Hydrogen-bond geometry (Å, °)

*D*—H⋯*A*	*D*—H	H⋯*A*	*D*⋯*A*	*D*—H⋯*A*
C1—H1⋯O4^i^	0.93	2.33	3.210 (3)	158
C4—H4⋯O3^ii^	0.93	2.56	3.412 (3)	153
C6—H6*B*⋯O3^iii^	0.96	2.57	3.470 (4)	157
C8—H8⋯O3^ii^	0.93	2.55	3.418 (3)	156

Symmetry codes: (i) 
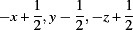
; (ii) 
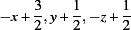
; (iii) 
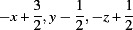
.

**Fig. 1.**
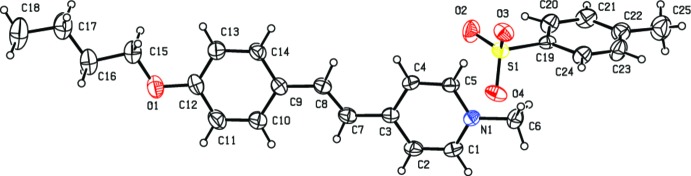
The molecular structure of (I), with 30% probability displacement ellipsoids for non-H atoms.

**Fig. 2.**
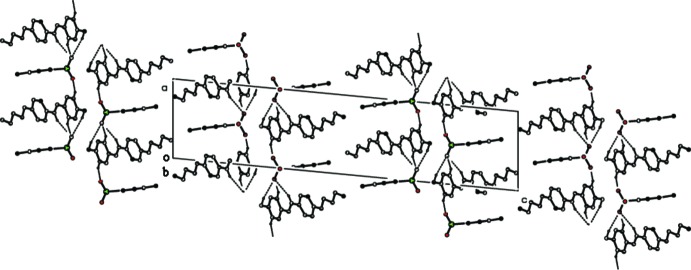
The packing of (I), viewed down [010]. Hydrogen bonds are shown as dashed lines. H atoms not involved in hydrogen bonding have been omitted.

## supplementary crystallographic information

### Comment

In continuation of our studies of molecular compounds with potential non-linear optical properties, which could be used in optoelectronic and photonic devices (Nalwa & Miyata, 1997), we herewith report the crystal structure of the title compound, (I), (Fig. 1).

The geometric parameters of the cation and anion in (I) are comparable to those in previously reported structures (Krishnakumar *et al.*, 2012; Sivakumar *et al.*, 2012). The benzene ring and pyridinium ring makes a dihedral angle of 10.00 (9)°. in the cation.

In the crystal, the anions and cations are linked by weak C—H···O (Table 1 & Fig. 2) and π···π [Cg1···Cg2 (1/2+x,-1/2-y,1/2+z) = 3.7658 (17)Å; Cg1 and Cg2 are the centroids of the N1/C1-C5 and C9-C14 rings, respectively] interactions.

### Experimental

The 4-butoxy-4'-N'-methyl stilbazolium tosylate was synthesized by the condensation reaction. The stoichiometric amount of reagents 4-picoline (4.65 g, 5 mmol), methyl p-toluenesulfonate (9.31 g, 5 mmol), and p-butoxybenzaldehyde (8.64 ml, 5 mmol) were refluxed 20 hours in the presence of piperidine. Colourless blocks of (I) were grown by slow evaporation of a methanol solution.

### Refinement

H atoms were positioned geometrically and refined using riding model with C-H = 0.93 Å and Uiso(H) = 1.2Ueq(C) for CH, C-H = 0.97 Å and Uiso(H) = 1.2Ueq(C) for CH_2_, C-H = 0.96 Å and Uiso(H) = 1.5Ueq(C) for CH_3_. H atoms for C20 and C23 were found from the difference Fourier map and C20-H20 and C23-H23 distances were restrained to 0.93 (1)Å.

### Crystal data

**Table:**

C_18_H_22_NO^+^·C_7_H_7_O_3_S^−^	*F*(000) = 936
*M**_r_* = 439.55	*D*_x_ = 1.255 Mg m^−^^3^
Monoclinic, *P*2_1_/*n*	Mo *K*α radiation, λ = 0.71073 Å
Hall symbol: -P 2yn	Cell parameters from 4259 reflections
*a* = 9.0884 (6) Å	θ = 2.0–28.3°
*b* = 6.4559 (5) Å	µ = 0.17 mm^−^^1^
*c* = 39.827 (3) Å	*T* = 295 K
β = 95.404 (3)°	Block, colourless
*V* = 2326.4 (2) Å^3^	0.28 × 0.24 × 0.20 mm
*Z* = 4	

### Data collection

**Table:**

Bruker Kappa APEXII CCD diffractometer	5629 independent reflections
Radiation source: fine-focus sealed tube	4258 reflections with *I* > 2σ(*I*)
Graphite monochromator	*R*_int_ = 0.028
ω and φ scan	θ_max_ = 28.3°, θ_min_ = 2.1°
Absorption correction: multi-scan (*SADABS*; Sheldrick, 1996)	*h* = −12→12
*T*_min_ = 0.954, *T*_max_ = 0.967	*k* = −8→8
20608 measured reflections	*l* = −53→53

### Refinement

**Table:**

Refinement on *F*^2^	Primary atom site location: structure-invariant direct methods
Least-squares matrix: full	Secondary atom site location: difference Fourier map
*R*[*F*^2^ > 2σ(*F*^2^)] = 0.069	Hydrogen site location: inferred from neighbouring sites
*wR*(*F*^2^) = 0.160	H atoms treated by a mixture of independent and constrained refinement
*S* = 1.16	*w* = 1/[σ^2^(*F*_o_^2^) + (0.0359*P*)^2^ + 2.0379*P*] where *P* = (*F*_o_^2^ + 2*F*_c_^2^)/3
5629 reflections	(Δ/σ)_max_ < 0.001
291 parameters	Δρ_max_ = 0.23 e Å^−^^3^
2 restraints	Δρ_min_ = −0.39 e Å^−^^3^

### Special details

**Table:**

Geometry. All esds (except the esd in the dihedral angle between two l.s. planes) are estimated using the full covariance matrix. The cell esds are taken into account individually in the estimation of esds in distances, angles and torsion angles; correlations between esds in cell parameters are only used when they are defined by crystal symmetry. An approximate (isotropic) treatment of cell esds is used for estimating esds involving l.s. planes.
Refinement. Refinement of F^2^ against ALL reflections. The weighted R-factor wR and goodness of fit S are based on F^2^, conventional R-factors R are based on F, with F set to zero for negative F^2^. The threshold expression of F^2^ > 2sigma(F^2^) is used only for calculating R-factors(gt) etc. and is not relevant to the choice of reflections for refinement. R-factors based on F^2^ are statistically about twice as large as those based on F, and R- factors based on ALL data will be even larger.

### Fractional atomic coordinates and isotropic or equivalent isotropic displacement parameters (Å^2^)

**Table:**

	*x*	*y*	*z*	*U*_iso_*/*U*_eq_	
S1	0.50367 (7)	0.31025 (11)	0.192843 (17)	0.04861 (19)	
O1	0.5412 (3)	1.3940 (4)	0.42984 (6)	0.0809 (7)	
O2	0.5079 (3)	0.5330 (3)	0.19010 (6)	0.0789 (7)	
O3	0.6435 (2)	0.2208 (4)	0.20606 (5)	0.0640 (6)	
O4	0.3804 (2)	0.2348 (4)	0.20972 (5)	0.0664 (6)	
N1	0.4977 (2)	0.0292 (3)	0.27689 (5)	0.0469 (5)	
C1	0.3694 (3)	0.0849 (5)	0.28821 (7)	0.0573 (7)	
H1	0.2863	0.0023	0.2833	0.069*	
C2	0.3587 (3)	0.2596 (5)	0.30663 (7)	0.0575 (7)	
H2	0.2683	0.2937	0.3144	0.069*	
C3	0.4789 (3)	0.3888 (4)	0.31426 (6)	0.0466 (6)	
C4	0.6110 (3)	0.3277 (5)	0.30151 (7)	0.0503 (6)	
H4	0.6949	0.4097	0.3055	0.060*	
C5	0.6177 (3)	0.1500 (4)	0.28339 (7)	0.0500 (6)	
H5	0.7066	0.1114	0.2754	0.060*	
C6	0.5076 (4)	−0.1583 (5)	0.25612 (7)	0.0612 (8)	
H6A	0.4267	−0.2491	0.2596	0.092*	
H6B	0.5993	−0.2279	0.2625	0.092*	
H6C	0.5031	−0.1200	0.2328	0.092*	
C7	0.4653 (3)	0.5724 (5)	0.33512 (7)	0.0556 (7)	
H7	0.3736	0.5967	0.3429	0.067*	
C8	0.5707 (3)	0.7068 (5)	0.34388 (7)	0.0519 (7)	
H8	0.6614	0.6843	0.3354	0.062*	
C9	0.5600 (3)	0.8877 (5)	0.36552 (6)	0.0497 (6)	
C10	0.4379 (3)	0.9313 (6)	0.38288 (8)	0.0700 (9)	
H10	0.3564	0.8437	0.3803	0.084*	
C11	0.4343 (4)	1.0995 (6)	0.40360 (9)	0.0765 (10)	
H11	0.3508	1.1248	0.4148	0.092*	
C12	0.5539 (3)	1.2327 (5)	0.40816 (7)	0.0607 (8)	
C13	0.6756 (3)	1.1942 (5)	0.39102 (8)	0.0653 (8)	
H13	0.7565	1.2829	0.3935	0.078*	
C14	0.6773 (3)	1.0237 (5)	0.37012 (7)	0.0580 (7)	
H14	0.7605	0.9996	0.3587	0.070*	
C15	0.6659 (4)	1.5244 (6)	0.43681 (8)	0.0738 (9)	
H15A	0.6903	1.5913	0.4162	0.089*	
H15B	0.7504	1.4430	0.4457	0.089*	
C16	0.6299 (5)	1.6859 (6)	0.46230 (9)	0.0851 (11)	
H16A	0.5430	1.7627	0.4535	0.102*	
H16B	0.6073	1.6175	0.4829	0.102*	
C17	0.7561 (6)	1.8339 (7)	0.47022 (10)	0.1014 (14)	
H17A	0.7817	1.8957	0.4493	0.122*	
H17B	0.8414	1.7567	0.4799	0.122*	
C18	0.7232 (7)	2.0047 (8)	0.49432 (12)	0.133 (2)	
H18A	0.6429	2.0875	0.4844	0.199*	
H18B	0.8093	2.0900	0.4989	0.199*	
H18C	0.6967	1.9451	0.5150	0.199*	
C19	0.4720 (2)	0.2172 (4)	0.15089 (6)	0.0428 (6)	
C20	0.4479 (4)	0.3501 (5)	0.12436 (8)	0.0613 (8)	
C21	0.4179 (4)	0.2789 (6)	0.09167 (8)	0.0700 (9)	
H21	0.4026	0.3738	0.0741	0.084*	
C22	0.4105 (4)	0.0713 (6)	0.08467 (8)	0.0687 (9)	
C23	0.4372 (4)	−0.0616 (5)	0.11138 (9)	0.0737 (10)	
C24	0.4687 (3)	0.0078 (5)	0.14422 (8)	0.0597 (8)	
H24	0.4875	−0.0869	0.1617	0.072*	
C25	0.3715 (5)	−0.0092 (8)	0.04919 (10)	0.1049 (14)	
H25A	0.4179	−0.1412	0.0467	0.157*	
H25B	0.4056	0.0868	0.0332	0.157*	
H25C	0.2663	−0.0243	0.0451	0.157*	
H23	0.435 (4)	−0.2033 (19)	0.1073 (8)	0.083 (11)*	
H20	0.449 (3)	0.489 (2)	0.1308 (8)	0.071 (10)*	

### Atomic displacement parameters (Å^2^)

**Table:**

	*U*^11^	*U*^22^	*U*^33^	*U*^12^	*U*^13^	*U*^23^
S1	0.0408 (3)	0.0486 (4)	0.0569 (4)	0.0088 (3)	0.0075 (3)	0.0052 (3)
O1	0.0891 (17)	0.0787 (17)	0.0752 (15)	0.0093 (14)	0.0099 (12)	−0.0245 (13)
O2	0.1106 (19)	0.0453 (13)	0.0813 (15)	0.0024 (13)	0.0112 (13)	−0.0055 (11)
O3	0.0428 (10)	0.0851 (16)	0.0633 (12)	0.0141 (10)	0.0009 (8)	0.0020 (11)
O4	0.0463 (10)	0.0839 (16)	0.0715 (13)	0.0134 (11)	0.0189 (9)	0.0157 (12)
N1	0.0499 (12)	0.0433 (13)	0.0469 (11)	−0.0035 (10)	0.0009 (9)	0.0074 (10)
C1	0.0428 (14)	0.0621 (19)	0.0664 (17)	−0.0118 (14)	0.0022 (12)	0.0051 (16)
C2	0.0379 (13)	0.069 (2)	0.0667 (17)	−0.0022 (13)	0.0094 (12)	0.0010 (16)
C3	0.0433 (13)	0.0479 (15)	0.0486 (13)	0.0010 (12)	0.0046 (10)	0.0079 (12)
C4	0.0405 (12)	0.0525 (16)	0.0580 (15)	−0.0096 (12)	0.0058 (11)	−0.0002 (14)
C5	0.0413 (13)	0.0511 (17)	0.0586 (15)	−0.0037 (12)	0.0104 (11)	0.0030 (13)
C6	0.078 (2)	0.0452 (16)	0.0594 (16)	−0.0041 (15)	−0.0014 (14)	−0.0017 (14)
C7	0.0431 (14)	0.0617 (19)	0.0630 (16)	0.0058 (14)	0.0108 (12)	0.0020 (15)
C8	0.0455 (13)	0.0559 (17)	0.0548 (15)	0.0068 (13)	0.0072 (11)	0.0052 (14)
C9	0.0495 (14)	0.0526 (17)	0.0469 (13)	0.0107 (13)	0.0044 (11)	0.0026 (13)
C10	0.0547 (17)	0.080 (2)	0.077 (2)	−0.0043 (17)	0.0136 (15)	−0.0155 (19)
C11	0.0638 (19)	0.091 (3)	0.077 (2)	0.0096 (19)	0.0193 (16)	−0.020 (2)
C12	0.0690 (19)	0.061 (2)	0.0519 (15)	0.0182 (16)	0.0064 (13)	−0.0063 (14)
C13	0.0618 (17)	0.066 (2)	0.0682 (18)	−0.0005 (16)	0.0069 (14)	−0.0090 (17)
C14	0.0527 (16)	0.0624 (19)	0.0605 (16)	0.0072 (14)	0.0129 (13)	−0.0060 (15)
C15	0.095 (3)	0.062 (2)	0.0630 (19)	0.009 (2)	0.0010 (17)	−0.0061 (17)
C16	0.123 (3)	0.069 (2)	0.0623 (19)	0.018 (2)	0.000 (2)	−0.0104 (18)
C17	0.154 (4)	0.078 (3)	0.070 (2)	−0.001 (3)	0.001 (2)	−0.010 (2)
C18	0.208 (6)	0.091 (3)	0.095 (3)	0.000 (4)	−0.008 (3)	−0.029 (3)
C19	0.0328 (11)	0.0383 (14)	0.0576 (14)	0.0006 (10)	0.0062 (10)	0.0095 (12)
C20	0.0727 (19)	0.0432 (17)	0.0667 (18)	0.0019 (15)	−0.0003 (15)	0.0114 (15)
C21	0.080 (2)	0.067 (2)	0.0604 (18)	−0.0054 (18)	−0.0040 (15)	0.0238 (17)
C22	0.069 (2)	0.071 (2)	0.0651 (19)	−0.0089 (18)	0.0039 (15)	−0.0003 (18)
C23	0.097 (3)	0.0449 (19)	0.077 (2)	−0.0071 (18)	−0.0009 (19)	−0.0015 (17)
C24	0.0704 (19)	0.0421 (16)	0.0659 (18)	−0.0007 (14)	0.0022 (14)	0.0145 (14)
C25	0.133 (4)	0.104 (3)	0.075 (2)	−0.023 (3)	−0.003 (2)	−0.011 (2)

### Geometric parameters (Å, º)

**Table:**

S1—O2	1.443 (2)	C12—C13	1.376 (4)
S1—O4	1.445 (2)	C13—C14	1.381 (4)
S1—O3	1.4487 (19)	C13—H13	0.9300
S1—C19	1.773 (3)	C14—H14	0.9300
O1—C12	1.364 (4)	C15—C16	1.512 (5)
O1—C15	1.418 (4)	C15—H15A	0.9700
N1—C1	1.339 (3)	C15—H15B	0.9700
N1—C5	1.346 (3)	C16—C17	1.503 (6)
N1—C6	1.474 (4)	C16—H16A	0.9700
C1—C2	1.354 (4)	C16—H16B	0.9700
C1—H1	0.9300	C17—C18	1.510 (6)
C2—C3	1.385 (4)	C17—H17A	0.9700
C2—H2	0.9300	C17—H17B	0.9700
C3—C4	1.404 (4)	C18—H18A	0.9600
C3—C7	1.459 (4)	C18—H18B	0.9600
C4—C5	1.360 (4)	C18—H18C	0.9600
C4—H4	0.9300	C19—C20	1.363 (4)
C5—H5	0.9300	C19—C24	1.378 (4)
C6—H6A	0.9600	C20—C21	1.383 (5)
C6—H6B	0.9600	C20—H20	0.933 (10)
C6—H6C	0.9600	C21—C22	1.369 (5)
C7—C8	1.315 (4)	C21—H21	0.9300
C7—H7	0.9300	C22—C23	1.370 (5)
C8—C9	1.460 (4)	C22—C25	1.516 (5)
C8—H8	0.9300	C23—C24	1.386 (5)
C9—C14	1.379 (4)	C23—H23	0.930 (10)
C9—C10	1.391 (4)	C24—H24	0.9300
C10—C11	1.366 (5)	C25—H25A	0.9600
C10—H10	0.9300	C25—H25B	0.9600
C11—C12	1.384 (5)	C25—H25C	0.9600
C11—H11	0.9300		
			
O2—S1—O4	113.47 (14)	C9—C14—C13	122.3 (3)
O2—S1—O3	113.34 (15)	C9—C14—H14	118.8
O4—S1—O3	112.88 (13)	C13—C14—H14	118.8
O2—S1—C19	105.64 (13)	O1—C15—C16	108.8 (3)
O4—S1—C19	105.08 (12)	O1—C15—H15A	109.9
O3—S1—C19	105.44 (12)	C16—C15—H15A	109.9
C12—O1—C15	117.6 (3)	O1—C15—H15B	109.9
C1—N1—C5	119.7 (2)	C16—C15—H15B	109.9
C1—N1—C6	120.7 (2)	H15A—C15—H15B	108.3
C5—N1—C6	119.5 (2)	C17—C16—C15	111.7 (4)
N1—C1—C2	121.0 (3)	C17—C16—H16A	109.3
N1—C1—H1	119.5	C15—C16—H16A	109.3
C2—C1—H1	119.5	C17—C16—H16B	109.3
C1—C2—C3	121.7 (3)	C15—C16—H16B	109.3
C1—C2—H2	119.2	H16A—C16—H16B	107.9
C3—C2—H2	119.2	C16—C17—C18	113.9 (4)
C2—C3—C4	115.8 (3)	C16—C17—H17A	108.8
C2—C3—C7	120.5 (2)	C18—C17—H17A	108.8
C4—C3—C7	123.7 (2)	C16—C17—H17B	108.8
C5—C4—C3	120.8 (2)	C18—C17—H17B	108.8
C5—C4—H4	119.6	H17A—C17—H17B	107.7
C3—C4—H4	119.6	C17—C18—H18A	109.5
N1—C5—C4	121.0 (2)	C17—C18—H18B	109.5
N1—C5—H5	119.5	H18A—C18—H18B	109.5
C4—C5—H5	119.5	C17—C18—H18C	109.5
N1—C6—H6A	109.5	H18A—C18—H18C	109.5
N1—C6—H6B	109.5	H18B—C18—H18C	109.5
H6A—C6—H6B	109.5	C20—C19—C24	118.0 (3)
N1—C6—H6C	109.5	C20—C19—S1	121.1 (2)
H6A—C6—H6C	109.5	C24—C19—S1	120.9 (2)
H6B—C6—H6C	109.5	C19—C20—C21	121.6 (3)
C8—C7—C3	126.0 (3)	C19—C20—H20	113 (2)
C8—C7—H7	117.0	C21—C20—H20	125 (2)
C3—C7—H7	117.0	C22—C21—C20	121.2 (3)
C7—C8—C9	126.6 (3)	C22—C21—H21	119.4
C7—C8—H8	116.7	C20—C21—H21	119.4
C9—C8—H8	116.7	C21—C22—C23	116.9 (3)
C14—C9—C10	116.6 (3)	C21—C22—C25	121.9 (3)
C14—C9—C8	119.6 (2)	C23—C22—C25	121.2 (4)
C10—C9—C8	123.8 (3)	C22—C23—C24	122.4 (3)
C11—C10—C9	121.8 (3)	C22—C23—H23	119 (2)
C11—C10—H10	119.1	C24—C23—H23	119 (2)
C9—C10—H10	119.1	C19—C24—C23	119.8 (3)
C10—C11—C12	120.7 (3)	C19—C24—H24	120.1
C10—C11—H11	119.7	C23—C24—H24	120.1
C12—C11—H11	119.7	C22—C25—H25A	109.5
O1—C12—C13	124.9 (3)	C22—C25—H25B	109.5
O1—C12—C11	116.4 (3)	H25A—C25—H25B	109.5
C13—C12—C11	118.7 (3)	C22—C25—H25C	109.5
C12—C13—C14	119.9 (3)	H25A—C25—H25C	109.5
C12—C13—H13	120.1	H25B—C25—H25C	109.5
C14—C13—H13	120.1		
			
C5—N1—C1—C2	−1.1 (4)	C11—C12—C13—C14	−0.9 (5)
C6—N1—C1—C2	−178.4 (3)	C10—C9—C14—C13	0.5 (4)
N1—C1—C2—C3	0.7 (5)	C8—C9—C14—C13	−178.1 (3)
C1—C2—C3—C4	0.4 (4)	C12—C13—C14—C9	0.1 (5)
C1—C2—C3—C7	−177.9 (3)	C12—O1—C15—C16	−177.7 (3)
C2—C3—C4—C5	−1.1 (4)	O1—C15—C16—C17	−178.4 (3)
C7—C3—C4—C5	177.2 (3)	C15—C16—C17—C18	177.2 (3)
C1—N1—C5—C4	0.4 (4)	O2—S1—C19—C20	−4.2 (3)
C6—N1—C5—C4	177.8 (2)	O4—S1—C19—C20	116.1 (2)
C3—C4—C5—N1	0.7 (4)	O3—S1—C19—C20	−124.5 (2)
C2—C3—C7—C8	−179.6 (3)	O2—S1—C19—C24	176.9 (2)
C4—C3—C7—C8	2.2 (5)	O4—S1—C19—C24	−62.8 (2)
C3—C7—C8—C9	−178.1 (3)	O3—S1—C19—C24	56.7 (3)
C7—C8—C9—C14	−174.7 (3)	C24—C19—C20—C21	1.3 (5)
C7—C8—C9—C10	6.8 (5)	S1—C19—C20—C21	−177.7 (3)
C14—C9—C10—C11	−0.4 (5)	C19—C20—C21—C22	0.4 (5)
C8—C9—C10—C11	178.2 (3)	C20—C21—C22—C23	−1.4 (5)
C9—C10—C11—C12	−0.3 (6)	C20—C21—C22—C25	177.4 (3)
C15—O1—C12—C13	−4.1 (5)	C21—C22—C23—C24	0.8 (6)
C15—O1—C12—C11	175.6 (3)	C25—C22—C23—C24	−178.0 (4)
C10—C11—C12—O1	−178.8 (3)	C20—C19—C24—C23	−1.8 (5)
C10—C11—C12—C13	1.0 (5)	S1—C19—C24—C23	177.1 (3)
O1—C12—C13—C14	178.8 (3)	C22—C23—C24—C19	0.8 (5)

### Hydrogen-bond geometry (Å, º)

**Table:**

*D*—H···*A*	*D*—H	H···*A*	*D*···*A*	*D*—H···*A*
C1—H1···O4^i^	0.93	2.33	3.210 (3)	158
C4—H4···O3^ii^	0.93	2.56	3.412 (3)	153
C6—H6*B*···O3^iii^	0.96	2.57	3.470 (4)	157
C8—H8···O3^ii^	0.93	2.55	3.418 (3)	156

Symmetry codes: (i) −*x*+1/2, *y*−1/2, −*z*+1/2; (ii) −*x*+3/2, *y*+1/2, −*z*+1/2; (iii) −*x*+3/2, *y*−1/2, −*z*+1/2.
